# Tetra­butyl­ammonium bis­[4,4′-dimethyl-2,2′-(3,7-dimethyl-1*H*-4,2,1-benzothiaza­siline-1,1-di­yl)dibenzene­thiol­ato]vanadium(III) acetonitrile tetra­solvate

**DOI:** 10.1107/S1600536810022014

**Published:** 2010-06-26

**Authors:** Yi-Fang Tsai, Hua-Fen Hsu, Kuei-Fang Hsu, Ju-Chun Wang

**Affiliations:** a Department of Chemistry, National Cheng Kung University, Tainan 701, Taiwan; b Department of Chemistry, Soochow University, Taipei, Taiwan

## Abstract

In the title compound, [N(C_4_H_9_)_4_][V(C_23_H_21_NS_3_Si)_2_]·4CH_3_CN, the V^III^ atom (site symmetry 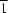) is coordinated by two *N*,*S*,*S*′-tridentate 4,4′-dimethyl-2,2′-(3,7-dimethyl-1*H*-4,2,1-benzothiaza­siline-1,1-di­yl)dibenzene­thiol­ate ligands in a distorted *trans*-VN_2_S_4_ octa­hedral geometry. The complete cation is generated by crystallographic twofold symmetry, with the V atom lying on the rotation axis. The unusual ligand arose from nucleophilic attack on the coordinated nitrile by the thiol­ate precursor and reduction of nitrile to the imidate.

## Related literature

For background to vanadium thiol­ate chemistry, see: Rehder (2008 ▶); Crans *et al.* (2004 ▶); Eady (2003 ▶); Janas & Sobota (2005 ▶); Ye *et al.* (2010 ▶); Tsai *et al.* (2007 ▶). For further mechanistic information, see: Block *et al.* (1989 ▶). For related structures, see: Zhu *et al.* (1997 ▶, 2002 ▶).
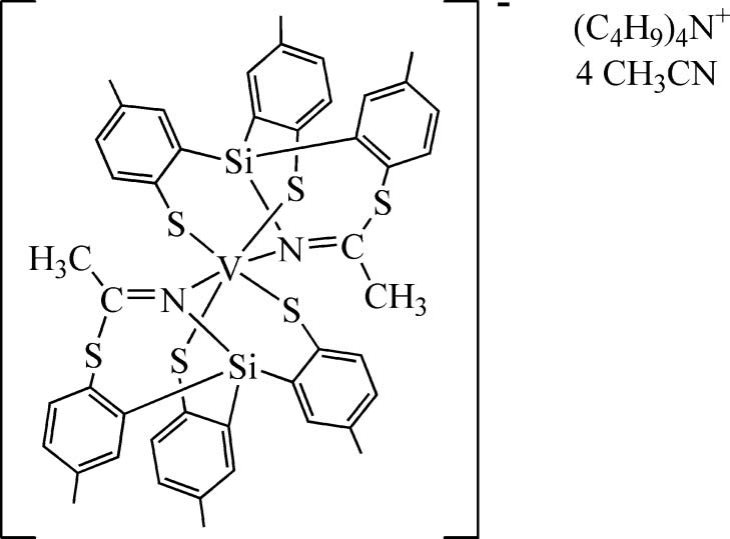


## Experimental

### 

#### Crystal data

(C_16_H_36_N)[V(C_23_H_21_NS_3_Si)_2_]·4C_2_H_3_N
*M* *_r_* = 1328.97
Monoclinic, 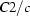

*a* = 27.0867 (16) Å
*b* = 14.6525 (9) Å
*c* = 22.0590 (13) Å
β = 126.359 (1)°
*V* = 7050.5 (7) Å^3^
*Z* = 4
Mo *K*α radiation
μ = 0.40 mm^−1^
*T* = 200 K
0.50 × 0.50 × 0.40 mm

#### Data collection

Bruker APEXII CCD area-detector diffractometer
Absorption correction: multi-scan (*SADABS*; Bruker, 2004 ▶) *T*_min_ = 0.490, *T*_max_ = 1.000
26980 measured reflections
8840 independent reflections
5635 reflections with *I* > 2σ(*I*)
*R*_int_ = 0.063

#### Refinement

*R*[*F* ^2^ > 2σ(*F* ^2^)] = 0.053
*wR*(*F* ^2^) = 0.144
*S* = 1.05
8840 reflections
396 parameters
H-atom parameters constrained
Δρ_max_ = 0.66 e Å^−3^
Δρ_min_ = −0.44 e Å^−3^

### 

Data collection: *APEX2* (Bruker, 2004 ▶); cell refinement: *SAINT* (Bruker, 2004 ▶); data reduction: *SAINT*; program(s) used to solve structure: *SHELXS97* (Sheldrick, 2008 ▶); program(s) used to refine structure: *SHELXL97* (Sheldrick, 2008 ▶); molecular graphics: *SHELXTL* (Sheldrick, 2008 ▶); software used to prepare material for publication: *SHELXTL*.

## Supplementary Material

Crystal structure: contains datablocks I, global. DOI: 10.1107/S1600536810022014/hb5425sup1.cif

Structure factors: contains datablocks I. DOI: 10.1107/S1600536810022014/hb5425Isup2.hkl

Additional supplementary materials:  crystallographic information; 3D view; checkCIF report

## Figures and Tables

**Table 1:** Selected geometric parameters (Å)

V1—N1	2.188 (2)
V1—S1	2.4161 (6)
V1—S2	2.4617 (7)

**Fig. 1.**
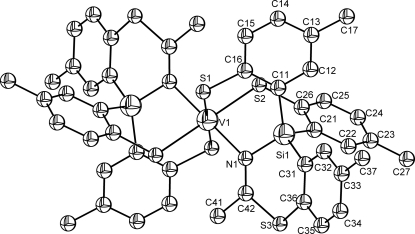
The anion in (I) with displacement ellipsoids drawn at the 35 % probability level. Unlabelled atoms are generated by the symmetry operation (1–x, –y, 1–z).

**Fig. 2.**
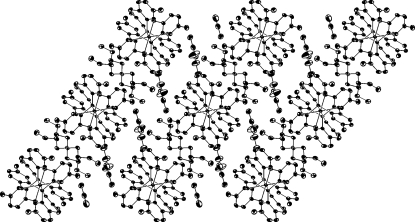
The packing diagram of (I): A view of the sheet parallel to the ac plane, H atoms have been omitted for clarity.

**Fig. 3.**
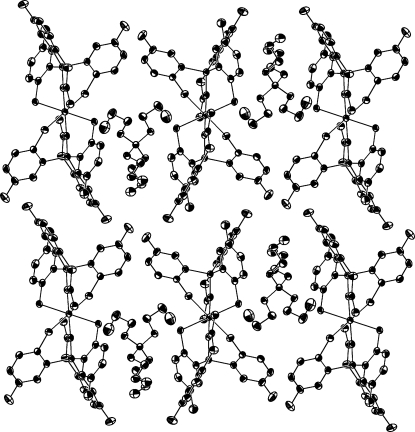
View of the packing in (I) approximately down the a axis, acetonitrile molecules and H atoms have been omitted for clarity.

## supplementary crystallographic information

### Comment

Vanadium thiolate chemistry has been drawing much attention due to its biological relevance as well as its medical application (Rehder, 2008; Crans *et al.*, 2004). For example, alternative nitrogenase is proposed to contain a [Fe_7_VS_9_] cofactor, where V site likely binds to three sulfides, His442 and homocitrate (Eady, 2003). To elucidate the role of vanadium in the enzyme, it is essential to understand fundamental chemistry of vanadium, particularly in a S-rich ligation environment (Janas & Sobota, 2005). Thus, we have been exploring the reactions of vanadium ion with thiolato containing ligands (Ye *et al.*, 2010; Tsai *et al.*, 2007). At this work, the reaction of [VCl_3_THF_3_] with H_3_L1 [H_3_L1 = HSi(5-Me–C_6_H_4_-2-SH)_3_] and three equivalents of nBu-Li in CH_3_CN generated a deep purple solution. The addition of the cation, [N(C_4_H_9_)_4_]Br, to the reaction mixture yielded a crystalline solid of the title compound (I).

The molecular structure of the anion in (I) is shown in Fig 1. It consists a V^III^ ion coordinated to two L2 ligands [L2 = Si{CH_3_(5-Me– C_6_H_4_-2-S)CN} (5-Me–C_6_H_4_-2-S)_2_]. L2 ligand has a S2N donor set that contains two benzenethiolates and one thioimidate group. The formation of a thioimidate group in L2 ligand upon the reaction is likely a consequence of nucleophilic attack on the coordinated nitrile by thiolate and reduction of nitrile to the imidate. Similar chemistry was demonstrated in a rhenium complex with thiolate ligands (Block *et al .*, 1989). The V^III^ ion lies on the inversion centre and forms a normal octahedral geometry with a S4N2 ligation environment, four S atoms from thiolate groups and two N atoms from thioimidate groups. Two N donor atoms of thioimidate groups are in *trans* positions.

The bond lengths and bond angles in compound (I) are shown in Table 1. The V—S distances of 2.416 (1) Å and 2.462 (1) Å are close to those of reported six-coordinate V^III^ thiolate complexes (Ye *et al.*, 2010; Zhu *et al.*, 2002; Zhu *et al.*, 1997).

The packing diagrams of compound (I) are shown in Fig 2. There is no interaction observed between molecules. The methyl groups on the phenyl rings of the ligands probably prevent the occurrence of inter-molecular π-π stacking interactions. The shortest distance between centers of two phenyl rings is 5.181 (2) Å.

### Experimental

A THF solution of VCl_3_(THF)_3_ (0.094 g, 0.25 mmol) was added to a acetonitrile solution (10 ml) of HSi(5-Me–C_6_H_4_-2-SH)_3_ (0.202 g, 0.51 mmol) and n-BuLi (0.098 g, 1.53 mmol) to generate a deep purple solution. The solution was concentrated and layered with [N(C_4_H_9_)_4_]Br (0.080 g, 0.25 mmol) in acetonitrile solution (5 ml). After one week, deep purple blocks of (I) were obtained.

### Refinement

H atoms were generated geometrically, with *C*—H_methyl_ = 0.96 Å; *C*—H_aryl_ = 0.93 Å; U_iso_H_methyl_ = 1.5U_eq_(C_methyl_); U_iso_H_aryl_ = 1.2U_eq_(C_aryl_). In case of the CH_3_ group, the positional parameters of the hydrogens were constrained by the SHELXL-97 command to the idealized tetrahedral geometry by the command AFIX 137 (Sheldrick, 2008).

### Crystal data

**Table:**

(C_16_H_36_N)[V(C_23_H_21_NS_3_Si)_2_]·4C_2_H_3_N	*F*(000) = 2824
*M**_r_* = 1328.97	*D*_x_ = 1.252 Mg m^−^^3^
Monoclinic, *C*2/*c*	Mo *K*α radiation, λ = 0.71073 Å
Hall symbol: -C 2yc	Cell parameters from 5232 reflections
*a* = 27.0867 (16) Å	θ = 2.3–28.1°
*b* = 14.6525 (9) Å	µ = 0.40 mm^−^^1^
*c* = 22.0590 (13) Å	*T* = 200 K
β = 126.359 (1)°	Block, deep purple
*V* = 7050.5 (7) Å^3^	0.50 × 0.50 × 0.40 mm
*Z* = 4	

### Data collection

**Table:**

Bruker APEXII CCD area-detector diffractometer	8840 independent reflections
Radiation source: fine-focus sealed tube	5635 reflections with *I* > 2σ(*I*)
graphite	*R*_int_ = 0.063
φ and ω scans	θ_max_ = 28.4°, θ_min_ = 1.7°
Absorption correction: multi-scan (*SADABS*; Bruker, 2004)	*h* = −33→36
*T*_min_ = 0.490, *T*_max_ = 1.000	*k* = −19→19
26980 measured reflections	*l* = −28→29

### Refinement

**Table:**

Refinement on *F*^2^	Primary atom site location: structure-invariant direct methods
Least-squares matrix: full	Secondary atom site location: difference Fourier map
*R*[*F*^2^ > 2σ(*F*^2^)] = 0.053	Hydrogen site location: inferred from neighbouring sites
*wR*(*F*^2^) = 0.144	H-atom parameters constrained
*S* = 1.05	*w* = 1/[σ^2^(*F*_o_^2^) + (0.0702*P*)^2^ + 1.1431*P*] where *P* = (*F*_o_^2^ + 2*F*_c_^2^)/3
8840 reflections	(Δ/σ)_max_ = 0.001
396 parameters	Δρ_max_ = 0.66 e Å^−^^3^
0 restraints	Δρ_min_ = −0.44 e Å^−^^3^
0 constraints	

### Special details

**Table:**

Geometry. All esds (except the esd in the dihedral angle between two l.s. planes) are estimated using the full covariance matrix. The cell esds are taken into account individually in the estimation of esds in distances, angles and torsion angles; correlations between esds in cell parameters are only used when they are defined by crystal symmetry. An approximate (isotropic) treatment of cell esds is used for estimating esds involving l.s. planes.
Refinement. Refinement of *F*^2^ against ALL reflections. The weighted *R*-factor wR and goodness of fit *S* are based on *F*^2^, conventional *R*-factors *R* are based on *F*, with *F* set to zero for negative *F*^2^. The threshold expression of *F*^2^ > σ(*F*^2^) is used only for calculating *R*-factors(gt) etc. and is not relevant to the choice of reflections for refinement. *R*-factors based on *F*^2^ are statistically about twice as large as those based on *F*, and *R*- factors based on ALL data will be even larger.

### Fractional atomic coordinates and isotropic or equivalent isotropic displacement parameters (Å^2^)

**Table:**

	*x*	*y*	*z*	*U*_iso_*/*U*_eq_	
V1	0.5000	0.0000	0.5000	0.02112 (15)	
Si1	0.47274 (3)	0.21699 (5)	0.46284 (4)	0.02117 (16)	
S1	0.57178 (3)	0.05741 (5)	0.47806 (4)	0.02504 (16)	
S2	0.53127 (3)	0.09662 (5)	0.60866 (4)	0.02710 (16)	
S3	0.32852 (3)	0.19171 (5)	0.31598 (4)	0.03403 (18)	
N1	0.43811 (10)	0.10819 (14)	0.42448 (12)	0.0222 (5)	
N2	0.5000	0.1548 (2)	0.2500	0.0255 (7)	
N3	0.2424 (3)	0.3201 (5)	0.3810 (3)	0.182 (4)	
N4	0.2660 (2)	0.5583 (4)	0.1270 (3)	0.125 (2)	
C11	0.55730 (12)	0.23091 (18)	0.51487 (14)	0.0237 (5)	
C12	0.58127 (12)	0.31516 (19)	0.55119 (15)	0.0271 (6)	
H12A	0.5547	0.3575	0.5489	0.033*	
C13	0.64280 (13)	0.3384 (2)	0.59036 (16)	0.0294 (6)	
C14	0.68221 (13)	0.2732 (2)	0.59387 (16)	0.0316 (6)	
H14A	0.7236	0.2868	0.6193	0.038*	
C15	0.66030 (13)	0.1887 (2)	0.55997 (16)	0.0298 (6)	
H15A	0.6874	0.1460	0.5637	0.036*	
C16	0.59806 (12)	0.16615 (18)	0.52012 (14)	0.0242 (5)	
C17	0.66594 (14)	0.4308 (2)	0.62743 (18)	0.0395 (7)	
H17A	0.6356	0.4595	0.6304	0.059*	
H17B	0.6737	0.4681	0.5982	0.059*	
H17C	0.7032	0.4232	0.6772	0.059*	
C21	0.45354 (12)	0.24289 (18)	0.52897 (15)	0.0232 (5)	
C22	0.41580 (12)	0.31461 (19)	0.52041 (16)	0.0274 (6)	
H22A	0.3971	0.3513	0.4778	0.033*	
C23	0.40540 (13)	0.3326 (2)	0.57407 (17)	0.0320 (7)	
C24	0.43478 (14)	0.2781 (2)	0.63787 (17)	0.0346 (7)	
H24A	0.4294	0.2902	0.6750	0.042*	
C25	0.47203 (14)	0.20590 (19)	0.64746 (16)	0.0308 (6)	
H25A	0.4908	0.1699	0.6905	0.037*	
C26	0.48153 (13)	0.18693 (18)	0.59321 (15)	0.0253 (6)	
C27	0.36400 (16)	0.4096 (2)	0.5628 (2)	0.0491 (9)	
H27A	0.3652	0.4161	0.6070	0.074*	
H27B	0.3228	0.3966	0.5202	0.074*	
H27C	0.3776	0.4653	0.5541	0.074*	
C31	0.43356 (12)	0.29874 (18)	0.38172 (15)	0.0235 (5)	
C32	0.46213 (13)	0.37586 (18)	0.37750 (16)	0.0276 (6)	
H32A	0.5037	0.3847	0.4153	0.033*	
C33	0.43175 (14)	0.43956 (19)	0.31998 (17)	0.0322 (6)	
C34	0.36973 (15)	0.4254 (2)	0.26367 (17)	0.0359 (7)	
H34A	0.3481	0.4678	0.2250	0.043*	
C35	0.33984 (14)	0.3497 (2)	0.26423 (16)	0.0336 (7)	
H35A	0.2985	0.3406	0.2256	0.040*	
C36	0.37176 (13)	0.28682 (19)	0.32289 (15)	0.0278 (6)	
C37	0.46425 (16)	0.5222 (2)	0.3187 (2)	0.0464 (8)	
H37A	0.5077	0.5119	0.3508	0.070*	
H37B	0.4547	0.5745	0.3362	0.070*	
H37C	0.4510	0.5329	0.2682	0.070*	
C41	0.34431 (13)	0.01434 (19)	0.34091 (16)	0.0304 (6)	
H41A	0.3674	−0.0339	0.3763	0.046*	
H41B	0.3359	−0.0011	0.2933	0.046*	
H41C	0.3064	0.0224	0.3348	0.046*	
C42	0.38056 (12)	0.10112 (18)	0.36956 (15)	0.0256 (6)	
C51	0.68030 (15)	0.3592 (2)	0.43581 (19)	0.0455 (8)	
H51A	0.6921	0.4068	0.4720	0.068*	
H51B	0.6849	0.3804	0.3983	0.068*	
H51C	0.7059	0.3068	0.4607	0.068*	
C52	0.61384 (14)	0.3336 (2)	0.39858 (17)	0.0337 (7)	
H52A	0.6092	0.3133	0.4368	0.040*	
H52B	0.5883	0.3871	0.3745	0.040*	
C53	0.59212 (15)	0.2588 (2)	0.34048 (17)	0.0363 (7)	
H53A	0.6235	0.2122	0.3602	0.044*	
H53B	0.5855	0.2840	0.2956	0.044*	
C54	0.53288 (13)	0.21578 (19)	0.31999 (15)	0.0289 (6)	
H54A	0.5051	0.2642	0.3119	0.035*	
H54B	0.5420	0.1797	0.3624	0.035*	
C55	0.31405 (18)	0.0422 (3)	0.1459 (2)	0.0661 (11)	
H55A	0.2820	0.0107	0.1438	0.099*	
H55B	0.3003	0.0574	0.0958	0.099*	
H55C	0.3243	0.0972	0.1748	0.099*	
C56	0.36886 (16)	−0.0172 (2)	0.18187 (19)	0.0450 (8)	
H56A	0.3578	−0.0732	0.1531	0.054*	
H56B	0.3819	−0.0332	0.2320	0.054*	
C57	0.42201 (15)	0.0271 (2)	0.18751 (18)	0.0384 (7)	
H57A	0.4069	0.0578	0.1405	0.046*	
H57B	0.4507	−0.0197	0.1956	0.046*	
C58	0.45487 (14)	0.09565 (19)	0.25166 (16)	0.0302 (6)	
H58A	0.4247	0.1346	0.2490	0.036*	
H58B	0.4766	0.0630	0.2991	0.036*	
C61	0.1622 (2)	0.2502 (4)	0.2518 (2)	0.0830 (15)	
H61A	0.1721	0.2621	0.2173	0.124*	
H61B	0.1229	0.2761	0.2324	0.124*	
H61C	0.1611	0.1855	0.2577	0.124*	
C62	0.2073 (3)	0.2900 (4)	0.3223 (3)	0.106 (2)	
C63	0.18371 (17)	0.6328 (3)	0.0028 (2)	0.0598 (10)	
H63A	0.1721	0.6895	0.0129	0.090*	
H63B	0.1487	0.5934	−0.0250	0.090*	
H63C	0.1990	0.6443	−0.0261	0.090*	
C64	0.22959 (19)	0.5909 (3)	0.0711 (3)	0.0625 (11)	

### Atomic displacement parameters (Å^2^)

**Table:**

	*U*^11^	*U*^22^	*U*^33^	*U*^12^	*U*^13^	*U*^23^
V1	0.0260 (3)	0.0229 (3)	0.0141 (3)	0.0032 (3)	0.0117 (3)	0.0016 (2)
Si1	0.0233 (4)	0.0242 (4)	0.0162 (3)	0.0026 (3)	0.0117 (3)	0.0012 (3)
S1	0.0306 (4)	0.0265 (3)	0.0223 (3)	0.0039 (3)	0.0181 (3)	0.0017 (3)
S2	0.0351 (4)	0.0260 (4)	0.0160 (3)	0.0032 (3)	0.0129 (3)	0.0003 (3)
S3	0.0243 (4)	0.0312 (4)	0.0345 (4)	0.0040 (3)	0.0108 (3)	0.0033 (3)
N1	0.0252 (12)	0.0269 (12)	0.0144 (10)	0.0021 (9)	0.0116 (10)	0.0003 (9)
N2	0.0369 (19)	0.0229 (16)	0.0215 (16)	0.000	0.0198 (15)	0.000
N3	0.119 (5)	0.178 (6)	0.110 (4)	0.088 (4)	−0.009 (4)	−0.069 (4)
N4	0.099 (3)	0.198 (6)	0.102 (4)	0.087 (4)	0.073 (3)	0.093 (4)
C11	0.0256 (14)	0.0292 (14)	0.0162 (13)	0.0025 (11)	0.0124 (11)	0.0034 (11)
C12	0.0289 (15)	0.0309 (15)	0.0209 (13)	0.0012 (11)	0.0144 (12)	0.0000 (11)
C13	0.0311 (15)	0.0346 (16)	0.0215 (14)	−0.0039 (12)	0.0151 (13)	−0.0008 (12)
C14	0.0260 (15)	0.0408 (17)	0.0239 (14)	−0.0023 (12)	0.0126 (13)	0.0037 (13)
C15	0.0288 (15)	0.0338 (16)	0.0263 (15)	0.0077 (12)	0.0160 (13)	0.0083 (12)
C16	0.0266 (14)	0.0298 (14)	0.0160 (13)	0.0022 (11)	0.0126 (12)	0.0041 (11)
C17	0.0369 (18)	0.0411 (18)	0.0369 (18)	−0.0092 (14)	0.0199 (15)	−0.0101 (15)
C21	0.0239 (14)	0.0263 (14)	0.0209 (13)	−0.0046 (10)	0.0141 (12)	−0.0034 (11)
C22	0.0271 (14)	0.0278 (14)	0.0275 (15)	−0.0012 (11)	0.0162 (13)	−0.0039 (12)
C23	0.0341 (16)	0.0327 (16)	0.0370 (17)	−0.0072 (12)	0.0253 (15)	−0.0137 (13)
C24	0.0466 (19)	0.0355 (16)	0.0354 (17)	−0.0084 (13)	0.0319 (16)	−0.0125 (13)
C25	0.0436 (17)	0.0271 (15)	0.0258 (15)	−0.0093 (12)	0.0229 (14)	−0.0054 (12)
C26	0.0322 (15)	0.0238 (14)	0.0238 (14)	−0.0052 (11)	0.0186 (13)	−0.0060 (11)
C27	0.050 (2)	0.050 (2)	0.056 (2)	0.0040 (16)	0.036 (2)	−0.0153 (18)
C31	0.0277 (14)	0.0261 (14)	0.0188 (13)	0.0050 (11)	0.0149 (12)	0.0001 (11)
C32	0.0318 (15)	0.0284 (15)	0.0248 (14)	0.0047 (11)	0.0179 (13)	0.0002 (11)
C33	0.0439 (18)	0.0263 (15)	0.0272 (15)	0.0069 (13)	0.0215 (14)	0.0028 (12)
C34	0.0473 (19)	0.0285 (16)	0.0236 (15)	0.0116 (13)	0.0164 (15)	0.0064 (12)
C35	0.0324 (16)	0.0293 (16)	0.0239 (15)	0.0079 (12)	0.0084 (13)	0.0001 (12)
C36	0.0319 (15)	0.0270 (14)	0.0232 (14)	0.0052 (11)	0.0156 (13)	0.0004 (11)
C37	0.052 (2)	0.0323 (17)	0.051 (2)	0.0036 (15)	0.0284 (18)	0.0137 (16)
C41	0.0319 (16)	0.0314 (16)	0.0246 (15)	−0.0008 (12)	0.0149 (13)	−0.0018 (12)
C42	0.0286 (15)	0.0304 (15)	0.0192 (13)	0.0030 (11)	0.0149 (12)	−0.0006 (11)
C51	0.047 (2)	0.0407 (19)	0.0371 (18)	0.0006 (15)	0.0188 (17)	0.0009 (15)
C52	0.0411 (18)	0.0249 (15)	0.0301 (16)	−0.0016 (12)	0.0184 (15)	−0.0026 (12)
C53	0.0438 (18)	0.0357 (17)	0.0312 (16)	−0.0070 (13)	0.0233 (15)	−0.0050 (13)
C54	0.0394 (17)	0.0278 (15)	0.0221 (14)	0.0005 (12)	0.0196 (13)	−0.0033 (11)
C55	0.049 (2)	0.084 (3)	0.060 (3)	−0.003 (2)	0.030 (2)	0.006 (2)
C56	0.052 (2)	0.051 (2)	0.0305 (17)	−0.0151 (16)	0.0235 (17)	−0.0018 (15)
C57	0.053 (2)	0.0337 (16)	0.0347 (17)	−0.0107 (14)	0.0294 (17)	−0.0064 (14)
C58	0.0423 (17)	0.0268 (14)	0.0285 (15)	−0.0020 (12)	0.0248 (14)	0.0015 (12)
C61	0.064 (3)	0.118 (4)	0.039 (2)	0.026 (3)	0.015 (2)	−0.011 (3)
C62	0.080 (4)	0.124 (5)	0.062 (3)	0.056 (3)	0.014 (3)	−0.027 (3)
C63	0.049 (2)	0.065 (3)	0.057 (2)	0.0072 (19)	0.027 (2)	0.011 (2)
C64	0.058 (3)	0.081 (3)	0.061 (3)	0.027 (2)	0.042 (2)	0.027 (2)

### Geometric parameters (Å, °)

**Table:**

V1—N1^i^	2.188 (2)	C31—C32	1.404 (4)
V1—N1	2.188 (2)	C32—C33	1.386 (4)
V1—S1^i^	2.4161 (6)	C32—H32A	0.9300
V1—S1	2.4161 (6)	C33—C34	1.391 (4)
V1—S2	2.4617 (7)	C33—C37	1.508 (4)
V1—S2^i^	2.4617 (7)	C34—C35	1.378 (4)
Si1—N1	1.788 (2)	C34—H34A	0.9300
Si1—C21	1.855 (3)	C35—C36	1.395 (4)
Si1—C11	1.868 (3)	C35—H35A	0.9300
Si1—C31	1.874 (3)	C37—H37A	0.9600
S1—C16	1.767 (3)	C37—H37B	0.9600
S2—C26	1.771 (3)	C37—H37C	0.9600
S3—C36	1.768 (3)	C41—C42	1.498 (4)
S3—C42	1.781 (3)	C41—H41A	0.9600
N1—C42	1.292 (3)	C41—H41B	0.9600
N2—C58	1.516 (3)	C41—H41C	0.9600
N2—C58^ii^	1.516 (3)	C51—C52	1.518 (4)
N2—C54	1.532 (3)	C51—H51A	0.9600
N2—C54^ii^	1.532 (3)	C51—H51B	0.9600
N3—C62	1.148 (6)	C51—H51C	0.9600
N4—C64	1.131 (5)	C52—C53	1.515 (4)
C11—C12	1.404 (4)	C52—H52A	0.9700
C11—C16	1.407 (4)	C52—H52B	0.9700
C12—C13	1.390 (4)	C53—C54	1.522 (4)
C12—H12A	0.9300	C53—H53A	0.9700
C13—C14	1.400 (4)	C53—H53B	0.9700
C13—C17	1.511 (4)	C54—H54A	0.9700
C14—C15	1.386 (4)	C54—H54B	0.9700
C14—H14A	0.9300	C55—C56	1.482 (5)
C15—C16	1.402 (4)	C55—H55A	0.9600
C15—H15A	0.9300	C55—H55B	0.9600
C17—H17A	0.9600	C55—H55C	0.9600
C17—H17B	0.9600	C56—C57	1.515 (4)
C17—H17C	0.9600	C56—H56A	0.9700
C21—C22	1.399 (4)	C56—H56B	0.9700
C21—C26	1.407 (4)	C57—C58	1.520 (4)
C22—C23	1.395 (4)	C57—H57A	0.9700
C22—H22A	0.9300	C57—H57B	0.9700
C23—C24	1.387 (4)	C58—H58A	0.9700
C23—C27	1.504 (4)	C58—H58B	0.9700
C24—C25	1.389 (4)	C61—C62	1.413 (7)
C24—H24A	0.9300	C61—H61A	0.9600
C25—C26	1.393 (4)	C61—H61B	0.9600
C25—H25A	0.9300	C61—H61C	0.9600
C27—H27A	0.9600	C63—C64	1.404 (5)
C27—H27B	0.9600	C63—H63A	0.9600
C27—H27C	0.9600	C63—H63B	0.9600
C31—C36	1.393 (4)	C63—H63C	0.9600
			
N1^i^—V1—N1	180.0	C32—C33—C37	121.5 (3)
N1^i^—V1—S1^i^	86.54 (6)	C34—C33—C37	120.8 (3)
N1—V1—S1^i^	93.46 (6)	C35—C34—C33	121.1 (3)
N1^i^—V1—S1	93.46 (6)	C35—C34—H34A	119.4
N1—V1—S1	86.54 (6)	C33—C34—H34A	119.4
S1^i^—V1—S1	180.0	C34—C35—C36	119.7 (3)
N1^i^—V1—S2	90.53 (6)	C34—C35—H35A	120.1
N1—V1—S2	89.47 (6)	C36—C35—H35A	120.1
S1^i^—V1—S2	81.86 (2)	C31—C36—C35	121.5 (3)
S1—V1—S2	98.14 (2)	C31—C36—S3	123.2 (2)
N1^i^—V1—S2^i^	89.47 (6)	C35—C36—S3	115.2 (2)
N1—V1—S2^i^	90.53 (6)	C33—C37—H37A	109.5
S1^i^—V1—S2^i^	98.14 (2)	C33—C37—H37B	109.5
S1—V1—S2^i^	81.86 (2)	H37A—C37—H37B	109.5
S2—V1—S2^i^	180.0	C33—C37—H37C	109.5
N1—Si1—C21	103.98 (11)	H37A—C37—H37C	109.5
N1—Si1—C11	119.77 (11)	H37B—C37—H37C	109.5
C21—Si1—C11	107.92 (12)	C42—C41—H41A	109.5
N1—Si1—C31	106.07 (11)	C42—C41—H41B	109.5
C21—Si1—C31	110.86 (12)	H41A—C41—H41B	109.5
C11—Si1—C31	108.12 (12)	C42—C41—H41C	109.5
C16—S1—V1	109.28 (8)	H41A—C41—H41C	109.5
C26—S2—V1	117.31 (9)	H41B—C41—H41C	109.5
C36—S3—C42	107.89 (13)	N1—C42—C41	126.0 (2)
C42—N1—Si1	121.20 (19)	N1—C42—S3	127.1 (2)
C42—N1—V1	127.58 (18)	C41—C42—S3	106.85 (19)
Si1—N1—V1	109.60 (11)	C52—C51—H51A	109.5
C58—N2—C58^ii^	110.3 (3)	C52—C51—H51B	109.5
C58—N2—C54	107.73 (15)	H51A—C51—H51B	109.5
C58^ii^—N2—C54	111.25 (15)	C52—C51—H51C	109.5
C58—N2—C54^ii^	111.25 (15)	H51A—C51—H51C	109.5
C58^ii^—N2—C54^ii^	107.73 (15)	H51B—C51—H51C	109.5
C54—N2—C54^ii^	108.6 (3)	C53—C52—C51	112.2 (3)
C12—C11—C16	118.1 (2)	C53—C52—H52A	109.2
C12—C11—Si1	115.51 (19)	C51—C52—H52A	109.2
C16—C11—Si1	126.4 (2)	C53—C52—H52B	109.2
C13—C12—C11	123.3 (3)	C51—C52—H52B	109.2
C13—C12—H12A	118.4	H52A—C52—H52B	107.9
C11—C12—H12A	118.4	C52—C53—C54	111.3 (2)
C12—C13—C14	117.4 (3)	C52—C53—H53A	109.4
C12—C13—C17	121.0 (3)	C54—C53—H53A	109.4
C14—C13—C17	121.5 (3)	C52—C53—H53B	109.4
C15—C14—C13	120.8 (3)	C54—C53—H53B	109.4
C15—C14—H14A	119.6	H53A—C53—H53B	108.0
C13—C14—H14A	119.6	C53—C54—N2	114.9 (2)
C14—C15—C16	121.3 (3)	C53—C54—H54A	108.5
C14—C15—H15A	119.3	N2—C54—H54A	108.5
C16—C15—H15A	119.3	C53—C54—H54B	108.5
C15—C16—C11	119.1 (3)	N2—C54—H54B	108.5
C15—C16—S1	120.1 (2)	H54A—C54—H54B	107.5
C11—C16—S1	120.8 (2)	C56—C55—H55A	109.5
C13—C17—H17A	109.5	C56—C55—H55B	109.5
C13—C17—H17B	109.5	H55A—C55—H55B	109.5
H17A—C17—H17B	109.5	C56—C55—H55C	109.5
C13—C17—H17C	109.5	H55A—C55—H55C	109.5
H17A—C17—H17C	109.5	H55B—C55—H55C	109.5
H17B—C17—H17C	109.5	C55—C56—C57	113.2 (3)
C22—C21—C26	119.2 (2)	C55—C56—H56A	108.9
C22—C21—Si1	124.8 (2)	C57—C56—H56A	108.9
C26—C21—Si1	115.95 (19)	C55—C56—H56B	108.9
C23—C22—C21	121.8 (3)	C57—C56—H56B	108.9
C23—C22—H22A	119.1	H56A—C56—H56B	107.7
C21—C22—H22A	119.1	C56—C57—C58	111.3 (3)
C24—C23—C22	118.0 (3)	C56—C57—H57A	109.4
C24—C23—C27	121.5 (3)	C58—C57—H57A	109.4
C22—C23—C27	120.5 (3)	C56—C57—H57B	109.4
C23—C24—C25	121.4 (3)	C58—C57—H57B	109.4
C23—C24—H24A	119.3	H57A—C57—H57B	108.0
C25—C24—H24A	119.3	N2—C58—C57	113.0 (2)
C24—C25—C26	120.6 (3)	N2—C58—H58A	109.0
C24—C25—H25A	119.7	C57—C58—H58A	109.0
C26—C25—H25A	119.7	N2—C58—H58B	109.0
C25—C26—C21	119.0 (2)	C57—C58—H58B	109.0
C25—C26—S2	119.4 (2)	H58A—C58—H58B	107.8
C21—C26—S2	121.55 (19)	C62—C61—H61A	109.5
C23—C27—H27A	109.5	C62—C61—H61B	109.5
C23—C27—H27B	109.5	H61A—C61—H61B	109.5
H27A—C27—H27B	109.5	C62—C61—H61C	109.5
C23—C27—H27C	109.5	H61A—C61—H61C	109.5
H27A—C27—H27C	109.5	H61B—C61—H61C	109.5
H27B—C27—H27C	109.5	N3—C62—C61	176.4 (9)
C36—C31—C32	116.4 (2)	C64—C63—H63A	109.5
C36—C31—Si1	119.8 (2)	C64—C63—H63B	109.5
C32—C31—Si1	123.7 (2)	H63A—C63—H63B	109.5
C33—C32—C31	123.5 (3)	C64—C63—H63C	109.5
C33—C32—H32A	118.3	H63A—C63—H63C	109.5
C31—C32—H32A	118.3	H63B—C63—H63C	109.5
C32—C33—C34	117.7 (3)	N4—C64—C63	178.3 (5)

Symmetry codes: (i) −*x*+1, −*y*, −*z*+1; (ii) −*x*+1, *y*, −*z*+1/2.
